# Significance of Fractionated Administration of Thalidomide Combined With γ-Ray Irradiation in Terms of Local Tumor Response and Lung Metastasis

**DOI:** 10.14740/wjon855w

**Published:** 2014-08-25

**Authors:** Shin-ichiro Masunaga, Yu Sanada, Takahiro Moriwaki, Keizo Tano, Yoshinori Sakurai, Hiroki Tanaka, Minoru Suzuki, Natsuko Kondo, Masaru Narabayashi, Tsubasa Watanabe, Yosuke Nakagawa, Akira Maruhashi, Koji Ono

**Affiliations:** a Department of Radiation Life and Medical Science, Research Reactor Institute, Kyoto University, 2-1010, Asashiro-nishi, Kumatori-cho, Sennan-gun, Osaka 590-0494, Japan

**Keywords:** Thalidomide, Vascular normalization, Nicotinamide, Mild temperature hyperthermia, Quiescent cell, Acute hypoxia, Chronic hypoxia

## Abstract

**Background:**

The aim of this study was to evaluate the significance of fractionated administration of thalidomide combined with γ-ray irradiation in terms of local tumor response and lung metastatic potential, referring to the response of intratumor quiescent (Q) cells.

**Methods:**

B16-BL6 melanoma tumor-bearing C57BL/6 mice were continuously given 5-bromo-2’-deoxyuridine (BrdU) to label all proliferating (P) cells. The tumor-bearing mice then received γ-ray irradiation after thalidomide treatment through a single or two consecutive daily intraperitoneal administrations up to a total dose of 400 mg/kg in combination with an acute hypoxia-releasing agent (nicotinamide) or mild temperature hyperthermia (MTH). Immediately after the irradiation, cells from some tumors were isolated and incubated with a cytokinesis blocker. The responses of the Q and total (= P + Q) cell populations were assessed based on the frequency of micronuclei using immunofluorescence staining for BrdU. In other tumor-bearing mice, 17 days after irradiation, macroscopic lung metastases were enumerated.

**Results:**

Thalidomide raised the sensitivity of the total cell population more remarkably than Q cells in both single and daily administrations. Daily administration of thalidomide elevated the sensitivity of both the total and Q cell populations, but especially the total cell population, compared with single administration. Daily administration, especially combined with MTH, decreased the number of lung metastases.

**Conclusion:**

Daily fractionated administration of thalidomide in combination with γ-ray irradiation was thought to be more promising than single administration because of its potential to enhance local tumor response and repress lung metastatic potential.

## Introduction

It was believed that antiangiogenic therapy prevents tumor vascular growth and proliferation, thus depriving the tumor of the oxygen and nutrients necessary for survival [1]. Subsequent study, however, suggested that antiangiogenic therapy may also “normalize” the tumor vasculature for a short period of time, thereby providing a window of opportunity for improved drug delivery and enhanced sensitivity to radiation [1, 2]. The originally used approach relies on using agents that directly target vascular endothelial growth factor (VEGF) or VEGF receptor on endothelial cells. Another strategy is to indirectly target VEGF by inhibiting oncogenic signaling in cancer cells, hence decreasing both oncogenic activity and VEGF secretion [3, 4]. Inhibition of tyrosine kinase receptor signaling through RAS, phosphoinositide 3-kinase (PI-3K), and AKT was shown to result in enhanced vascular function, and this normalization enhanced tumor oxygenation and the delivery of cytotoxic drugs that might promote antitumor activity [3, 4].

Tumor hypoxia results from either limited oxygen diffusion (chronic hypoxia) or limited perfusion (acute hypoxia) [5]. Further, it was reported that acute and cyclic, but not chronic, hypoxia significantly increased the number of spontaneous lung metastases, and that this effect was due to the influence of acute hypoxia treatment on the primary tumor [6, 7].

Thalidomide has been reported to induce tumor blood vessel normalization in a mouse model [8, 9]. Today, thalidomide is being mainly applied as a treatment of certain cancers (multiple myeloma) and of a complication of leprosy. Here, using a readily metastasizing murine melanoma cell line, we tried to analyze the significance of combined treatment with thalidomide in radiotherapy with γ-rays in combination with an acute hypoxia-releasing agent nicotinamide or mild temperature hyperthermia (MTH), already shown to have the potential to release tumor cells from diffusion-limited chronic hypoxia [10, 11], in terms of local tumor response and lung metastatic potential. Concerning the local tumor response, the effect not only on the total (= proliferating (P) + quiescent (Q)) tumor cell population but also on the Q cell population was evaluated using our original method for selectively detecting the response of Q cells in solid tumors [12].

## Methods

### Mice and tumors

B16-BL6 murine melanoma cells (Institute of Development, Aging and Cancer, Tohoku University) derived from C57BL/6 mice were maintained *in vitro* in RPMI-1640 medium supplemented with 10% fetal bovine serum. Tumor cells (1.25 × 10^5^) were inoculated subcutaneously into the left hind leg of 8-week-old syngeneic female C57BL/6 mice (Japan Animal Co., Ltd, Osaka, Japan). Eighteen days later, the tumors, approximately 7 mm in diameter, were employed for γ-ray irradiation in this study, and the body weight of the tumor-bearing mice was 20.1 ± 2.3 g (mean ± standard error). Mice were handled according to the Recommendations for Handling of Laboratory Animals for Biomedical Research, compiled by the Committee on Safety Handling Regulations for Laboratory Animal Experiments at our university. The p53 of B16-BL6 tumor cells is the wild type [13].

### Labeling with 5-bromo-2’-deoxyuridine (BrdU)

Twelve days after the inoculation, mini-osmotic pumps (Durect Corporation, Cupertino, CA, USA) containing BrdU dissolved in physiological saline (250 mg/mL) were implanted subcutaneously into the animals’ backs for 6 days to label all P cells. The percentage of labeled cells after continuous labeling with BrdU was 54.3±6.1%, and reached a plateau at this stage. Therefore, tumor cells not incorporating BrdU after continuous labeling were regarded as Q cells.

### Treatment

After the labeling with BrdU, tumor-bearing mice received γ-ray irradiation. γ-ray irradiation was performed with a cobalt-60 γ-ray irradiator at a dose rate of 2.5 Gy/min with tumor-bearing mice held in a specially constructed device with the tail firmly fixed with an adhesive tape. Lead blocks were used to avoid irradiating body parts other than the tumor-bearing left hind leg.

Racemic thalidomide ((RS)*-*2-(2,6-dioxopiperidin-3-yl)-1H-isoindole-1,3(2H)-dione, C_13_H_10_N_2_O_4_, MW = 258.23) dissolved in dimethyl sulfoxide (DMSO, C_2_H_6_OS, MW = 78.13) was intraperitoneally administered to tumor-bearing mice 1 day before irradiation at a dose of 400 mg/kg, or two consecutive days before irradiation at a dose of 200 mg/kg (112 mg thalidomide/mL DMSO). Some tumor-bearing mice further received an intraperitoneal administration of nicotinamide (1,000 mg/kg) dissolved in physiological saline 1 h before the neutron irradiation. Others were subjected to local MTH at 40 °C for 60 min by immersing the implanted tumor in a water bath immediately before being irradiated [14]. Temperatures at the tumor’s center equilibrated within 3 - 4 min after immersion in the water bath and remained 0.2 - 0.3 °C below the bath’s temperature. The water bath’s temperature was maintained at 0.3 °C above the desired tumor temperature [14].

Each irradiation group also included mice that were not pretreated with BrdU.

### Immunofluorescence staining of BrdU-labeled cells and micronucleus (MN) assay

Immediately after irradiation, some tumors excised from the mice given BrdU were minced and trypsinized (0.05% trypsin and 0.02% ethylenediamine-tetraacetic acid (EDTA) in phosphate-buffered saline (PBS), 37 °C, 15 min). Tumor cell suspensions were incubated for 72 h in tissue culture dishes containing complete medium and 1.0 µg/mL of cytochalasin-B to inhibit cytokinesis while allowing nuclear division. The cultures were trypsinized, and cell suspensions were fixed and resuspended with cold Carnoy’s fixative (ethanol/acetic acid = 3:1 in volume). Each suspension was placed on a glass microscope slide, dried at room temperature and treated with 2 M hydrochloric acid for 60 min at room temperature to dissociate the histones and partially denature the DNA. The slides were immersed in borax-borate buffer (pH 8.5) to neutralize the acid. BrdU-labeled tumor cells were detected by indirect immunofluorescence staining using a monoclonal anti-BrdU antibody (Becton Dickinson, San Jose, CA, USA) and a fluorescein isothiocyanate (FITC)-conjugated antimouse IgG antibody (Sigma, St. Louis, MO, USA). To distinguish the tumor cells stained with green-emitting FITC and observe them separately, cells on the slides were treated with red-emitting propidium iodide (PI, 2 µg/mL in PBS) as a background staining and monitored under a fluorescence microscope.

When cell division is disrupted, or the chromosomes are broken or damaged by chemicals or radiation, then the distribution of genetic material between the two daughter nuclei during cell division is affected and pieces or entire chromosomes fail to be included in either of the two daughter nuclei. The genetic material that is not incorporated into a new nucleus forms an “MN”. Thus, the frequency of MN formation reflects the genotoxicity of a chemical compound and radiation very well. The MN frequency in cells not labeled with BrdU could be examined by counting the micronuclei in the binuclear cells that showed only red fluorescence. The MN frequency was defined as the ratio of the number of micronuclei in the binuclear cells to the total number of binuclear cells observed [12].

The ratios obtained in tumors not pretreated with BrdU indicated the MN frequency at all phases in the total tumor cell population. More than 300 binuclear cells were counted to determine the MN frequency.

### Clonogenic cell survival assay

The clonogenic cell survival assay was also performed for the implanted tumors in mice given no BrdU using an *in vivo*-*in vitro* assay method immediately after irradiation. The BrdU-unlabeled tumors were excised, weighed, minced, and disaggregated by stirring for 20 min at 37 °C in PBS containing 0.05% trypsin and 0.02% EDTA. The cell yield was 1.2 ± 0.4 × 10^7^/g tumor weight. Appropriate numbers of viable tumor cells from the single cell suspension were plated on 60- or 100-mm tissue culture dishes, and, 12 days later, colonies were fixed with ethanol, stained with Giemsa, and counted. For the tumors that received no irradiation, the plating efficiencies for the total tumor cell populations and the MN frequencies for the total and Q cell populations are shown in Table 1. The plating efficiency indicates the percentage of cells seeded that grew into colonies when the tumors received no irradiation. The fraction of cells surviving a given dose is determined by counting the number of macroscopic colonies as a fraction of the number of cells seeded, followed by allowance, that is, dividing by the plating efficiency.

**Table 1:** Surviving Fraction and Micronucleus Frequency at 0 Gy

	Without thalidomide	With thalidomide (once)	With thalidomide (twice)
Surviving fraction at 0 Gy (%)			
Without nicotinamide or mild temperature hyperthermia	84.4 ± 8.2^a^	60.0 ± 5.5	69.8 ± 6.3
With nicotinamide	81.4 ± 7.3	55.0 ± 4.3	60.0 ± 5.7
With mild temperature hyperthermia	83.5 ± 8.7	56.0 ± 5.9	60.5 ± 5.3
Micronucleus frequency at 0 Gy			
Total tumor cell population			
Without nicotinamide or mild temperature hyperthermia	0.050 ± 0.005	0.081 ± 0.008	0.070 ± 0.007
With nicotinamide	0.057 ± 0.006	0.090 ± 0.008	0.082 ± 0.008
With mild temperature hyperthermia	0.054 ± 0.005	0.085 ± 0.009	0.080 ± 0.008
Quiescent cells			
Without nicotinamide or mild temperature hyperthermia	0.077 ± 0.007	0.102 ± 0.01	0.094 ± 0.009
With nicotinamide	0.084 ± 0.008	0.11 ± 0.01	0.101 ± 0.01
With mild temperature hyperthermia	0.081 ± 0.008	0.103 ± 0.01	0.099 ± 0.009

^a^Mean ± standard error (n = 6). Q cells showed significantly higher micronucleus frequencies than the total cell population under each set of conditions (P < 0.05). Thalidomide administration resulted in significantly lower surviving fractions and significantly higher micronucleus frequencies in both the total and Q cell populations than absolutely no treatment (P < 0.05).

As stated above, the MN frequencies for Q cells were obtained from BrdU-unlabeled cells in tumors after continuous BrdU labeling *in vivo*. The MN frequencies and surviving fractions (SFs) for total tumor cell populations were obtained from cells in tumors not pretreated with BrdU. Thus, we could not detect any interaction between BrdU and irradiation in our data for the MN frequency and SF.

### Metastasis assessment

Seventeen days after irradiation (= 35 days after the inoculation of B16-BL6 melanoma cells), the tumor-bearing mice were killed by cervical dislocation, and their lungs were removed, briefly washed with distilled water, cleaned of extraneous tissue, fixed in Bouin’s solution overnight (Sigma), and stored in buffered formalin 10% (Sigma) until metastases were counted. Macroscopically visible metastases were counted under a dissection microscope [15]. Eighteen days after the inoculation and immediately before exposure to the neutron beam, macroscopic lung metastases were also counted as background data. The number was 7.5 ± 2.2.

### Data analysis and statistics

Three mice with a tumor in the left hind leg were used to assess each set of conditions and each experiment was repeated three times. Namely, nine mice were used for each set of conditions. To examine the differences between pairs of values, Student’s *t*-test was used when variances of the two groups could be assumed to be equal; otherwise the Welch *t*-test was used. P values are from two-sided tests. The data on cell survival and MN frequencies were fitted to the linear quadratic dose relationship [16].

## Results

Table 1 shows the SFs without γ-ray radiation for the total tumor cell population and the MN frequencies without γ-ray radiation for the total and Q cell populations. Q cells showed significantly higher MN frequencies at 0 Gy than the total cell population under each set of conditions (P < 0.05). Thalidomide administration produced significantly lower SFs and significantly higher MN frequencies at 0 Gy in both the total and Q cell populations than did absolutely no treatment (P < 0.05). The values at 0 Gy for both methods of administering thalidomide were almost the same although slightly lower SFs and higher MN frequencies were observed for the single administration than daily fractionated administration. Furthermore, the combined treatment with nicotinamide or MTH resulted in slightly lower SFs and significantly higher MN frequencies at 0 Gy in both the total and Q cell populations. The values at 0 Gy for the combined treatment with nicotinamide or MTH were almost the same although slightly lower SFs and higher MN frequencies were observed for nicotinamide than MTH.

**Figure 1:**
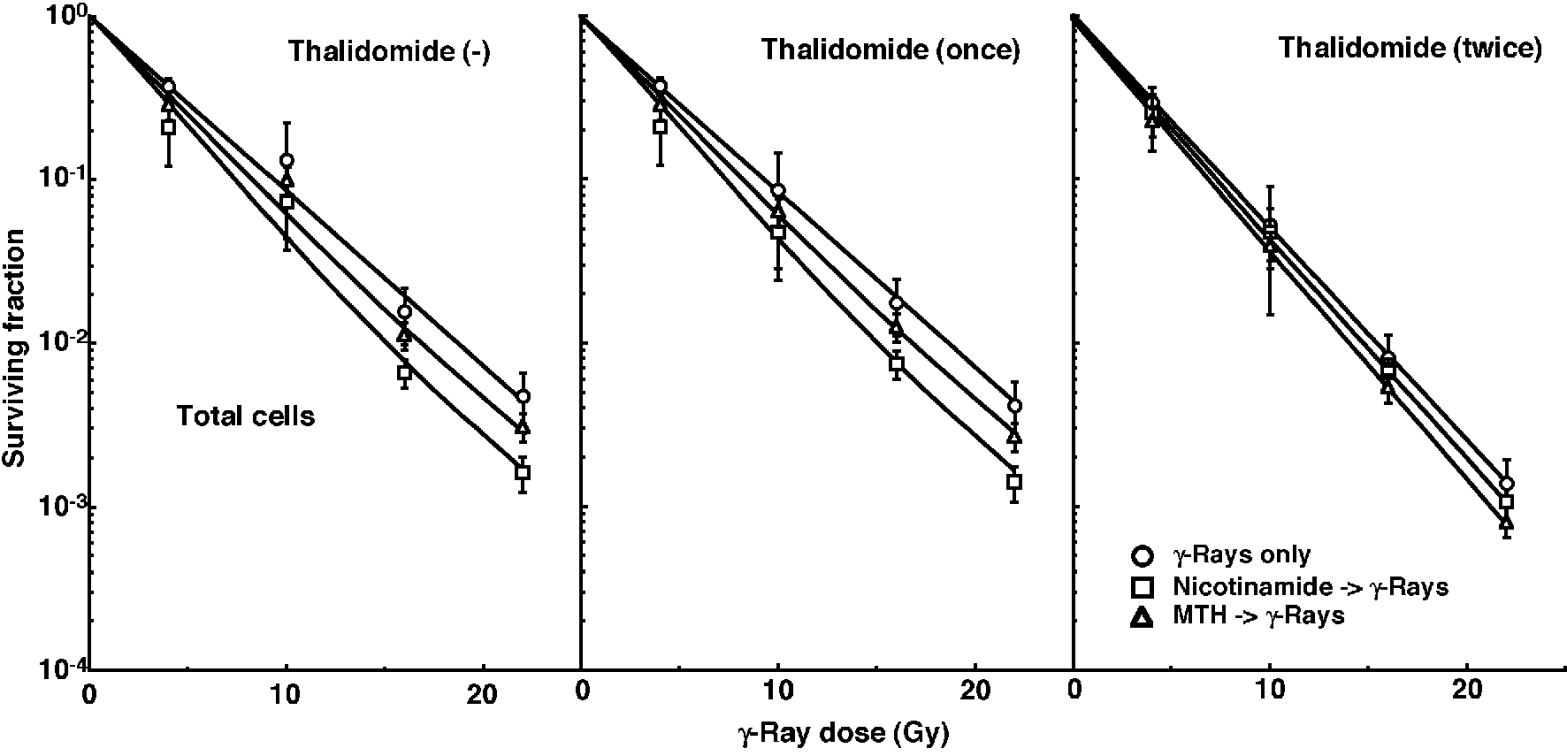
Cell survival curves for the total cell population from B16-BL6 tumors irradiated with γ-rays following single or two intraperitoneal administrations of thalidomide in combination with nicotinamide treatment or mild temperature hyperthermia (MTH) on day 18 after tumor cell inoculation. Circles: γ-ray irradiation only; squares: γ-ray irradiation with nicotinamide treatment; triangles: γ-ray irradiation with MTH. Bars represent standard errors (n = 6).

Cell survival curves for the total tumor cell population as a function of radiation dose are shown in Figure 1. The SFs without thalidomide and with singly administered thalidomide decreased in the following order: after γ-ray irradiation alone > after γ-ray irradiation following MTH > after γ-ray irradiation following nicotinamide administration. Meanwhile, the SFs with daily fractionated thalidomide decreased in the order: after γ-ray irradiation alone > after γ-ray irradiation following nicotinamide administration > after γ-ray irradiation following MTH.

**Figure 2:**
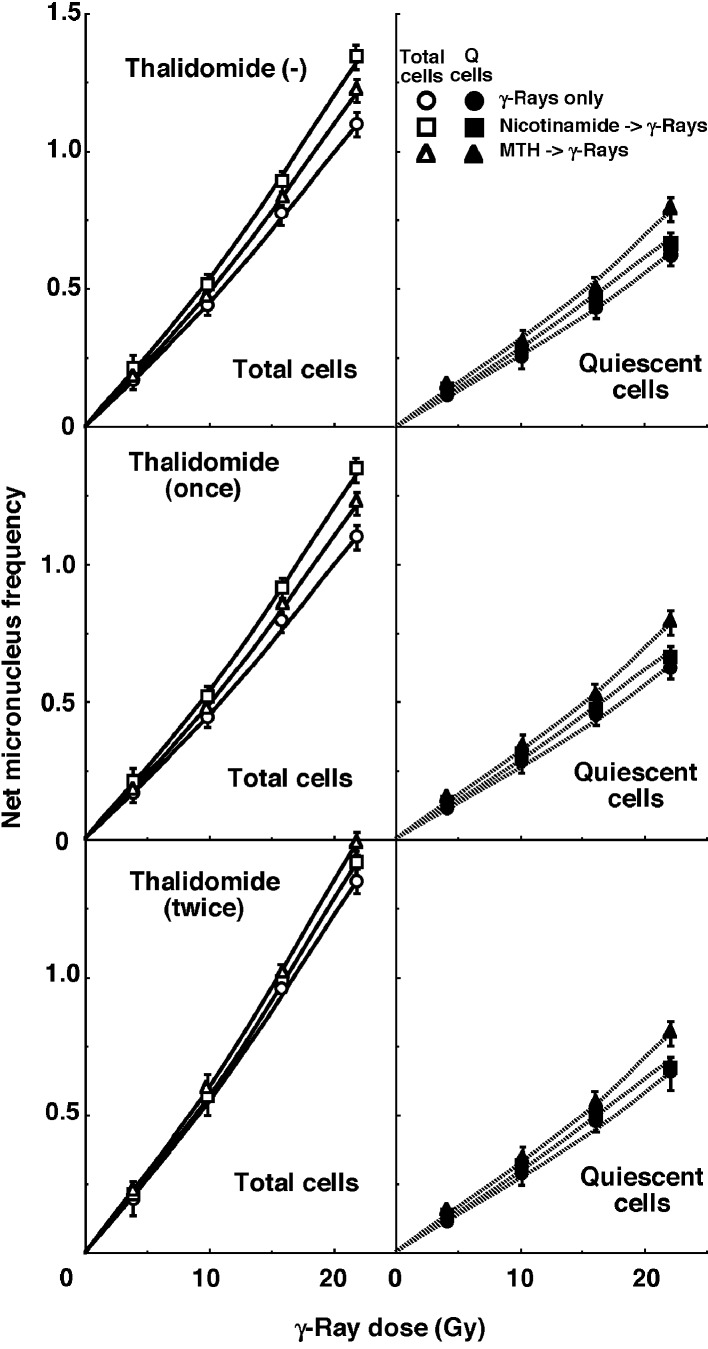
Dose response curves of the net micronucleus frequency for total (open symbols: left panels) and quiescent (solid symbols: right panels) cell populations from B16-BL6 tumors irradiated with γ-rays following single or two intraperitoneal administrations of thalidomide in combination with nicotinamide treatment or mild temperature hyperthermia (MTH) on day 18 after tumor cell inoculation. Circles: γ-ray irradiation only; squares: γ-ray irradiation with nicotinamide treatment; triangles: γ-ray irradiation with MTH. Bars represent standard errors (n = 6).

For baseline correction, we used the net MN frequency to exclude the MN frequency in non-irradiated tumors. The net MN frequency was defined as the MN frequency in the irradiated tumors minus that in the non-irradiated tumors. Dose response curves for the net MN frequency in total and Q tumor cell populations as a function of radiation dose are shown in Figure 2. Overall, the net MN frequencies were significantly lower in the Q cells than the total cell population (P < 0.05). In the total cell population treated without thalidomide or with singly administered thalidomide, the net MN frequency increased in the following order: after γ-ray irradiation alone < after γ-ray irradiation following MTH < after γ-ray irradiation following nicotinamide administration. In the total cell population with daily administered thalidomide and Q cell populations with or without thalidomide administration, the net MN frequency increased in the order: after γ-ray irradiation alone < after γ-ray irradiation following nicotinamide administration < after γ-ray irradiation following MTH.

To estimate the radio-enhancing effect of thalidomide, irradiation with thalidomide in both the total and Q cell populations was compared with irradiation only, using the data obtained without MTH or nicotinamide shown in Figures 1 and 2 (Table 2). The radio-enhancing effect of thalidomide was significantly larger than 1.0, and thalidomide enhanced the sensitivity of the total cell population slightly more than that of the Q cell population, especially for daily fractionated administration, without any significant differences. Further, daily fractionated administration increased the sensitivity of both cell populations more than single administration, especially for the total cell population, with significant differences.

**Table 2:** Enhancement Ratios^a^ Due to Combined Treatment With Thalidomide

	With thalidomide (once)	With thalidomide (twice)
Surviving fraction = 0.03		
Total cell population	1.0 ± 0.05^b, c^	1.25 ± 0.1^c^
Net micronucleus frequency = 0.6		
Total cell population	1.0 ± 0.05^d^	1.2 ± 0.1^d^
Quiescent cells	1.05 ± 0.1	1.1 ± 0.1

^a^The ratio of the dose of radiation necessary to obtain each end-point without thalidomide to that needed to obtain each end-point with thalidomide. ^b^ Mean ± standard error (n = 6). ^c, d^Significant differences between two values (P < 0.05).

To estimate the radio-enhancing effect of combined treatment with nicotinamide administration or MTH in both the total and Q cell populations, the data shown in Figures 1 and 2 were used (Table 3). Without thalidomide and with singly administered thalidomide, the combination with nicotinamide and MTH had more of an enhancing effect on the total and Q cell populations, respectively, without any significant differences. However, with daily fractionated administration of thalidomide, the enhancing effect of nicotinamide was reduced, resulting in a greater effect with MTH than nicotinamide on both the total and Q cell populations.

**Table 3:** Enhancement Ratios^a^ Due to Combined Treatment With Nicotinamide, or Mild Temperature Hyperthermia

	Nicotinamide	Mild temperature hyperthermia
Surviving fraction = 0.03		
Total cell population		
Without thalidomide	1.25 ± 0.1^b^	1.15 ± 0.05
With thalidomide (once)	1.25 ± 0.1	1.15 ± 0.05
With thalidomide (twice)	1.05 ± 0.05	1.1 ± 0.05
Net micronucleus frequency = 0.6		
Total cell population		
Without thalidomide	1.2 ± 0.1	1.05 ± 0.05
With thalidomide (once)	1.3 ± 0.1	1.05 ± 0.05
With thalidomide (twice)	1.05 ± 0.05	1.1 ± 0.05
Quiescent cells		
Without thalidomide	1.1 ± 0.05	1.2 ± 0.1
With thalidomide (once)	1.05 ± 0.05	1.15 ± 0.05
With thalidomide (twice)	1.1 ± 0.05	1.2 ± 0.05

^a^The ratio of the dose of radiation necessary to obtain each end-point without nicotinamide or mild temperature hyperthermia to that needed to obtain each end-point with nicotinamide or mild temperature hyperthermia. ^b^Mean ± standard error (n = 6).

To examine the difference in radio-sensitivity between the total and Q cell populations, dose-modifying factors were calculated using the data in Figures 1 and 2 (Table 4). All the values were significantly larger than 1.0. With thalidomide administration, the difference was slightly increased, especially with daily fractionated administration of thalidomide, without any significant differences. On further combined treatment with nicotinamide and MTH, the difference in radio-sensitivity seemed to be greater and smaller, respectively, although not significantly.

**Table 4:** Dose-Modifying Factors for Quiescent Cells Relative to the Total Tumor Cell Population^a^

	Without thalidomide	With thalidomide (once)	With thalidomide (twice)
Net micronucleus frequency = 0.6			
γ-Rays only	1.65 ± 0.15^b^	1.6 ± 0.1	1.9 ± 0.15
With nicotinamide	1.8 ± 0.15	2.0 ± 0.15	1.8 ± 0.15
With mild temperature hyperthermia	1.6 ± 0.15	1.55 ± 0.1	1.7 ± 0.1

^a^The ratio of the dose of radiation necessary to obtain each end-point in the quiescent cell population to that needed to obtain each end-point in the total tumor cell population. ^b^Mean ± standard error (n = 6).

**Figure 3:**
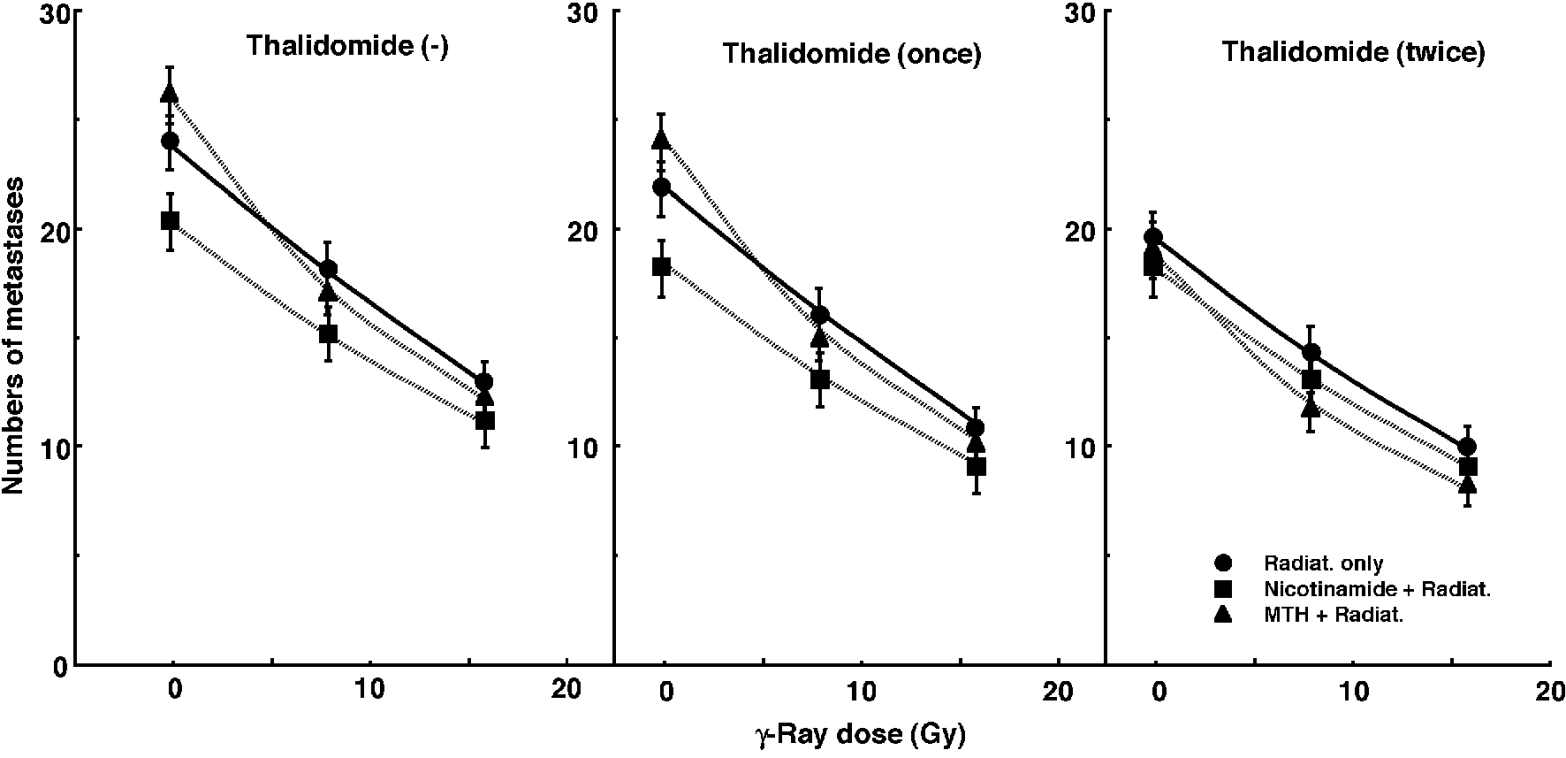
Counted numbers of macroscopic metastases in the lung on day 35 after tumor cell inoculation as a function of the dose of γ-ray irradiation following the single or two intraperitoneal administrations of thalidomide in combination with nicotinamide treatment or mild temperature hyperthermia (MTH) on day 18 after tumor cell inoculation. Circles: γ-ray irradiation only; squares: γ-ray irradiation with nicotinamide treatment; triangles: γ-ray irradiation with MTH. Bars represent standard errors (n = 6).

Figure 3 shows the numbers of lung metastases on day 35 after inoculation as a function of the absorbed dose of γ-ray irradiation with or without thalidomide administration in combination with nicotinamide administration or MTH. Without irradiation, the nicotinamide combination seemed to decrease the numbers of macroscopic metastases. With irradiation, as the absorbed dose increased, the numbers decreased. Without thalidomide and with singly administered thalidomide, the numbers decreased more remarkably with nicotinamide. However, with daily fractionated administration of thalidomide, the numbers decreased more remarkably with MTH. Namely, for no administration of thalidomide and the single administration of thalidomide, the combination with nicotinamide, and for the daily administration of thalidomide, the combination with MTH, which were most cytotoxic as an initial effect in a clonogenic cell survival assay, seemed to reduce the numbers of lung metastases from the local tumors most efficiently.

The numbers of lung metastases from local tumors that received irradiation under each set of conditions, which produced an identical SF of 0.6 as an initial effect (Fig. 1), were estimated using the data shown in Figure 3 (Table 5). Overall, the combination with thalidomide tended to decrease the numbers more than γ-ray irradiation only, especially with daily fractionated administration. The combination of nicotinamide without thalidomide or with singly administered thalidomide resulted in a slightly smaller number than without nicotinamide, although the difference was not significant. Further, the combination of MTH with daily administration of thalidomide led to a slightly smaller number than without MTH, though again not significantly. Thus, when thalidomide was administered daily combined with MTH, the number was significantly smaller than that for γ-ray irradiation only.

**Table 5:** The Numbers of Metastases From the Irradiated Tumors That Received Cytotoxic Treatment Producing a Similar Initial Local Effect^a^

	Without thalidomide	With thalidomide (once)	With thalidomide (twice)
Surviving fraction = 0.03			
γ-Rays only	14.1 ± 1.4^b, c, d, e^	12.2 ± 1.3	11.9 ± 1.2
With nicotinamide	13.1 ± 1.3	11.4 ± 1.2^c, f^	11.3 ± 1.2^d, g^
With mild temperature hyperthermia	14.1 ± 1.4^f, g, h^	12.2 ± 1.2	10.6 ± 1.1^e, h^

^a^Based on the data shown in Fig. 4, the estimated numbers of lung metastatic nodules from local tumors that received neutron beam irradiation with or without thalidomide in combination with nicotinamide or mild temperature hyperthermia, which produced an identical surviving fraction of 0.6 as an initial effect on Fig. 1. ^b^Mean ± standard error (n = 6). ^c-h^Significant differences between two values (P < 0.05).

## Discussion

Thalidomide was originally introduced as a non-barbiturate hypnotic, but withdrawn from the market due to teratogenic effects. However, it has been reintroduced and used for a number of immunological and inflammatory disorders due to its immunosuppressive and anti-angiogenic activity. It inhibits release of tumor necrosis factor from monocytes, and modulates other cytokine action. Thalidomide is racemic, and contains both left and right handed isomers in equal amounts: one enantiomer is effective against morning sickness, and the other is teratogenic. The enantiomers are converted to each other at physiological conditions (pH = 7.0) *in vivo*. That is, if a human is given D*-*thalidomide ((+)- or R*-*) or L*-*thalidomide ((-)- or S*-*), both isomers can be found in the serum. Hence, administering only one enantiomer will not prevent the teratogenic effect in humans. It is employed for the acute treatment of the cutaneous manifestations of moderate to severe erythema nodosum leprosum (ENL). Available data from *in vitro* studies and preliminary clinical trials suggest that its immunologic effects can vary substantially under different conditions, but may be related to suppression of excessive tumor necrosis factor production and down-modulation of selected cell surface adhesion molecules involved in leukocyte migration [17].

As a cancer treatment, thalidomide was shown to inhibit basic fibroblast growth factor (bFGF) as well as VEGF, two promoters of angiogenesis. It was reported to measure the modifications in the tumor environment early after an angiogenic treatment of thalidomide, with a special focus on possible normalization of the tumor vasculature that could be beneficial for radiotherapy [8]. Incidentally, angiogenesis also is critical during limb development of the foetus. Thalidomide directly inhibits angiogenesis induced by bFGF or VEGF *in vivo*. In 2009, it was confirmed that loss of newly formed blood vessels is the primary cause of thalidomide teratogenesis, and that developing limbs are particularly susceptible because of their relatively immature, highly angiogenic vessel network. Thus inhibition of angiogenesis is now thought to be a main mechanism of its teratogenicity [17, 18].

Tumor hypoxia can be manipulated by the treatment with an acute hypoxia-releasing agent, nicotinamide through its inhibiting action on temporary fluctuations in tumor blood flow [10, 11] or MTH, already shown to have the potential to release tumor cells from diffusion-limited chronic hypoxia [10, 11]. Taking into account our previous finding that the total and Q cell populations of B16-BL6 tumors are predominantly composed of acute and chronic hypoxia, respectively [11], it seems reasonable that the combination with nicotinamide and MTH had a greater enhancing effect on the total and Q cell populations, respectively, in the tumors treated without thalidomide or with singly administered thalidomide (Table 3). However, in the tumors treated daily administration of thalidomide, the effect of nicotinamide was reduced, leading to a greater enhancing effect of MTH than nicotinamide on both the total and Q cell populations (Table 3). Thus, it follows that the daily administration of thalidomide had already released cells from acute hypoxia before the nicotinamide treatment.

In our previous report, it was shown that the anti-VEGF agent bevacizumab has the potential to reduce perfusion-limited acute hypoxia during a vascular normalization window lasting some 2 - 5 days after the blocking of VEGF [19]. Irrespective of the same total dose of administered thalidomide, the single administration 1 day before irradiation could not release acute hypoxia, but daily fractionated administration before irradiation could. Therefore, it can be thought that the indirect targeting of VEGF by inhibiting oncogenic signaling decreased VEGF secretion, resulting in enhanced vascular function, and that this normalization enhanced tumor oxygenation including reducing acute hypoxia. During this normalization window induced by inhibiting oncogenic signaling, improvements in tumor oxygenation might promote antitumor activity [2-4, 8, 9]. Actually, even without the combined treatment with nicotinamide or MTH, the enhancing effect of thalidomide on both the total and Q cell populations was increased through daily fractionated administration (Table 2). In future, we would like to analyze this normalization window further.

The presence of Q cells is probably due, at least in part, to hypoxia and the depletion of nutrition, a consequence of poor vascular supply [20, 21]. As a result, Q cells are viable and clonogenic, but have ceased dividing. This might promote the formation of micronuclei at 0 Gy in Q tumor cells (Table 1). Q cells were shown to have significantly less radiosensitivity than the total cell population [12, 20, 21], that is, more Q cells survive radiotherapy than P cells (Fig. 2, Table 4). Thus, the control of chronic hypoxia-rich Q cells has a great impact on the outcome of conventional radiotherapy for controlling local tumors, resulting in the superiority of the combination with MTH in radiotherapy. As a result, the combined use of MTH led to a slight decrease in the difference in radiosensitivity (Table 4). In contrast, the combined use of nicotinamide which has an enhancing effect on the acute hypoxia-rich total cell population led to an increase in the difference in radiosensitivity. In addition, when thalidomide was further administered in a daily fractionated manner which also has the potential to release cells from acute hypoxia, the difference in radiosensitivity was also slightly increased.

Hypoxia is suggested to enhance metastasis by increasing genetic instability [6, 7]. Acute but not chronic hypoxia increased the number of macroscopic metastases in mouse lungs [6, 7]. We recently reported the significance of the administration of an acute hypoxia-releasing agent, nicotinamide, into tumor-bearing mice as a combined treatment with γ-ray irradiation in terms of repressing lung metastasis [11]. With or without irradiation, nicotinamide and the VEGF blocking treatment reduced the number of macroscopic lung metastases (Fig. 3, Table 5). During the window of vascular normalization, acute hypoxia may be released and this release is more important in suppressing metastasis from the primary tumor than is the release of cells from chronic hypoxia. Without irradiation, MTH slightly increased the number of metastases, implying that the release from chronic hypoxia is not as important in repressing metastasis as the release from acute hypoxia. However, hyperthermia is not thought to induce metastasis in the clinical setting [22]. Meanwhile, as the dose of γ-rays increased with irradiation, the number of macroscopic lung metastases decreased reflecting the decrease in the number of clonogenically viable tumor cells in the primary tumor (Fig. 3). The metastasis-repressing effect of the release from acute hypoxia without irradiation became less distinct with irradiation. This is partly because the metastasis-repressing effect achieved through a reduction in the number of clonogenic tumor cells by irradiation is much greater than that achieved by releasing tumor cells from acute hypoxia. An acute hypoxia-releasing treatment may be promising for reducing numbers of lung metastases.

### Conclusion

It was elucidated that control of the chronic hypoxia-rich Q cell population in primary solid tumors has the potential to impact the control of local tumors as a whole, while control of the acute hypoxia-rich total tumor cell population has the potential to impact the control of lung metastases. Namely, in conventional radiotherapy, daily fractionated administration of thalidomide combined with MTH is thought to have a great potential to control both local solid tumors and lung metastases from the local tumors.
